# Comorbidities Affecting Children with Autism Spectrum Disorder: A Retrospective Chart Review

**DOI:** 10.3390/children10081414

**Published:** 2023-08-19

**Authors:** Jessy Burns, Ryan Phung, Shayna McNeill, Ana Hanlon-Dearman, M. Florencia Ricci

**Affiliations:** 1 SSCY Centre, Department of Pediatrics and Child Health, University of Manitoba, Winnipeg, MB R3E 3G1, Canada; smcneill@rccinc.ca (S.M.); ahdearman@rccinc.ca (A.H.-D.); fricci@rccinc.ca (M.F.R.); 2 Department of Pediatrics and Child Health, University of Manitoba, Winnipeg, MB R3E 0Z3, Canada; rphung@manitoba-physicians.ca

**Keywords:** Autism spectrum disorder, global developmental delay, medical comorbidities, prematurity, neurological comorbidities, hypotonia, seizures, cerebral palsy, ASD resources

## Abstract

Autism spectrum disorder (ASD) is a developmental disorder characterized by deficits in social interaction/communication, restricted interests, and repetitive behaviors. Recent discussions have emerged worldwide regarding the heterogeneity around presentation/etiology and comorbidities. This study aimed to determine the frequency and characteristics of comorbidities among children diagnosed with ASD in Manitoba and to evaluate differences in presentation between those with and without medical comorbidities. We conducted a retrospective chart review of >1900 electronic charts at the only publicly funded referral site for children ≤6 years requiring evaluation for ASD in Manitoba. All children aged 0–6 years diagnosed with ASD at this site between May 2016 and September 2021 were identified. χ^2^ and *t*-tests were used to compare groups. Of the total of 1858 children identified, 1452 (78.1%) were boys, 251 (13.5%) were prematurely born, and 539 (29.0%) had ≥1 medical comorbidity. Global developmental delay (GDD) was diagnosed in 428 (23.0%). The age of referral and diagnosis did not differ between groups. Comorbidities were more common among premature children (16.0% vs. 12.5%, *p*: 0.005) and children with comorbid GDD (34.9% vs. 18.2%, *p* < 0.001). Neurological comorbidities were most common (37.1%). No sex difference in the overall presence of comorbidities was found (boys = 77.1% vs. 78.5%, *p*: 0.518); however, girls had a higher incidence of neurological comorbidities, e.g., cerebral palsy, seizures, hypotonia (14.8% vs. 9.64%, *p*: 0.009), as well as genetic comorbidities (4.92% vs. 2.75%, *p*: 0.04). The high rates of associated neurological conditions, GDD, and prematurity add heterogeneity to this group leading to potential difficulties with prognosis and service allocation. Primary vs. secondary ASD can be a way of separating individuals based on relevant medical comorbidities.

## 1. Introduction

Autism spectrum disorder (ASD) is a developmental disorder characterized by deficits in social interaction/communication, restricted interests, and repetitive behaviors [1]. It has been well documented that ASD rates are increasing worldwide [2]. Data from the United States Centers for Disease Control and Prevention (CDC) have reported ASD prevalence in 8-year-olds as 1 in 36 [3]. By comparison, in 2000, according to the CDC, this prevalence was 1 in 150 [3]. Despite a lot of research in this area, this marked increase has yet to be explained by one specific cause. Investigators have suggested several different explanations including increased public awareness of ASD, increased diagnostic availability and sensitivity [2], and increased heterogeneity.

Increased heterogeneity of those diagnosed with ASD has been occurring in tandem with increased prevalence [4,5]. As heterogeneity increases, and researchers are left comparing fundamentally dissimilar subjects with vastly different presentations of ASD, their ability to study the underlying neurocognitive mechanisms of ASD becomes much more complicated [6]. Some have proposed that it would be helpful to create “meaningful subgroups” of ASD [6], as although heterogeneity itself is not necessarily bad, the notion that ASD diagnosis can be “overly inclusive” has been pondered [7]. Heterogeneity may create challenges with regard to individualized patient care [8], as it contributes to complexity in presentation, diagnosis, and prognosis of ASD, especially as these relate to the accessibility of patient services/supports.

Many children who are diagnosed with ASD have comorbid neurodevelopmental disorders such as global developmental delay (GDD), attention deficit hyperactivity disorder (ADHD), and/or language/communication disorder. These are typically used to specify or qualify their diagnosis (such as ASD with accompanying GDD) [1]. In children diagnosed with ASD, there are also some who present with other medical comorbidities such as cerebral palsy, epilepsy, and genetic syndromes [9]. These children are arguably a “subgroup” of ASD as they may present somewhat differently than those with ASD who are otherwise healthy [8].

Do children with and without medical comorbidities have enough variability that classifying them differently is practical and meaningful? Should the use of “primary” and “secondary” ASD be pondered? The idea that patients with and without medical comorbidities can potentially be better classified, thus decreasing heterogeneity, is what led to the development of this study.

Therefore, the primary aim of this study was to determine the frequency and characteristics of medical comorbidities among children diagnosed with ASD at the Specialized Services for Children and Youth (SSCY) Centre—which is the only publicly funded referral site in the Canadian province of Manitoba for children ≤6 years requiring evaluation for ASD. Our secondary aim was to evaluate differences in presentation between those with and without medical comorbidities.

## 2. Materials and Methods

This study was a retrospective chart review of electronic charts of all children diagnosed with ASD at the Specialized Services for Children and Youth (SSCY) Centre.

The SSCY Centre is the only publicly funded referral site for children aged ≤6 years requiring evaluation for ASD in the Canadian province of Manitoba. There are seven developmental pediatricians who assess children and provide ASD diagnoses at the SSCY Centre. All seven have undergone the same medical and post-graduate specialty training. Diagnosis of ASD at the SSCY Centre is performed based on the Canadian Pediatric Society guidelines for ASD diagnosis, using “clinical judgment and DSM-5 criteria, with or without the data obtained using a diagnostic assessment tool”—e.g., Autism Diagnostic Observation Schedule—version 2 (ADOS-2): A standardized play-based test used in conjunction with clinical expertise to diagnose ASD, and consulting other professionals or a multidisciplinary team as indicated [10]. From both a clinical and a research perspective, this helps to unify our provincial diagnostic approach as well as access to services. Canada’s healthcare system is publicly funded, and although families may seek privately funded psychology assessment, the vast majority of ASD diagnosis in Manitoba is made through the public system at the SSCY Centre. This is important as it allows for reasonable inferences to be made about children with ASD province-wide based on the data collected in this study.

The inclusion criterion for this study was defined as follows: all children ≤6 years who were diagnosed with ASD at our center between May 2016 (the opening of a new clinic center) and September 2021 (when we began reviewing charts). The exclusion criteria were as follows: any child whose chart was missing information regarding diagnostic process and presence/absence of medical comorbidities. The chart review was conducted by the first and second authors, as well as two trained research nurses.

Ethics approval was obtained from the University of Manitoba Research Ethics Board, Shared Health and the Rehabilitation Centre for Children, ethics number H2021:147.

Variables of interest were identified as follows—child variables: sex (M/F); gestational age (term/premature as defined as birth <37 weeks gestational age); comorbid GDD (presence/absence); comorbid ADHD/suspected ADHD (presence/absence); medical comorbidities (presence/absence)—(see below); maternal/parental variables—maternal age at birth of the child (in years); education level (≥high school/<high school; high school defined as secondary school attended by teenagers ages 15–18); and diagnostic variables—age of the child at referral (in years), age of the child at diagnosis (in years), and use of the ADOS-2 diagnostic tool in a child’s diagnosis.

Medical comorbidities were identified based on the existence of conditions other than GDD (defined as delays in ≥2 domains of development, usually including the cognitive delay) [11], ADHD/suspected ADHD (defined as persistent pattern of inattention and/or hyperactivity interfering with functioning) [1], or language/communication disorders as these are common in children diagnosed with ASD and/or are used as specifiers and were studied as separate variables [1,12].

Specific medical conditions were further categorized as follows, based on common comorbidities in children with ASD as previously described in previous studies [8,9,13,14,15]: (1) neurological conditions, defined as any condition primarily originating from the nervous system, including cerebral palsy, brain injury, seizures, hypotonia, and macro-/microcephaly; (2) genetic conditions, defined as any condition primarily caused by a change in the individual’s genes; (3) congenital non-syndromic conditions, defined as any condition that an individual was born with but that has not been attributed to a specific syndrome or genetic change; (4) allergic conditions such as food/environmental allergy, asthma, and eczema; (5) gastrointestinal (GI) conditions defined as any condition originating in the GI system such as bowel obstruction, feeding difficulties, and gastroesophageal reflux; (6) ears, nose, and throat (ENT) conditions such as conductive hearing loss and recurrent acute otitis media; and (7) other conditions, defined as any otherwise unlisted conditions. Subjects were divided based on the presence or absence of 1 or more medical comorbidities.

Statistical analysis:

Descriptive statistics were run using SPSS statistical software – version 26. χ^2^ and *t*-tests were used to compare individuals with and without medical comorbidity as they related other variables (described above). Statistically significant differences between groups were noted with *p* values of <0.05.

## 3. Results

We reviewed a total of 1903 charts; a total of 1858 children were identified as meeting our inclusion criteria and were therefore included in this study. Of those identified, 1452 (78.1%) children were boys and 251 (13.5%) were prematurely born. Overall, out of the 1858 children with ASD, 23.0% had a formal diagnosis of GDD, and 5.76% had suspected or confirmed comorbid ADHD.

Subjects were divided based on the presence or absence of 1 or more medical comorbidities and compared in Table 1. Children who were prematurely born were found to have a statistically significant increased likelihood of medical comorbidity in addition to their diagnosis of ASD (16.0% vs. 12.5%, *p*: 0.005). Similarly, those with comorbid GDD also had a statistically significant increased likelihood of medical comorbidity (34.9% vs. 18.2%, *p*: < 0.001).

**Figure 1:**
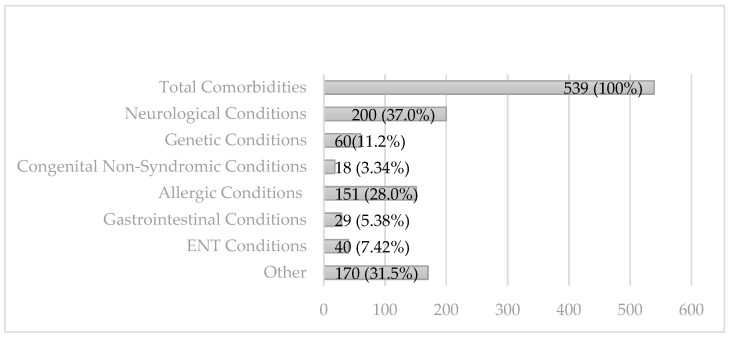
Comorbidities identified by category. NB percentages ≥100 as some individuals had more than one category of comorbidity.

A total of 539 children (29.0%) were identified to have 1 or more medical comorbidities. Comorbidities were then divided based on category (see Figure 1).

Overall rates of comorbidities in our subjects by category ranged from 1% (congenital non-syndromic conditions) to 10.8% (neurological conditions). Table 2 depicts all comorbidities existing in 2 or more children.

Children who were prematurely born were found to have a statistically significant increased likelihood of medical comorbidity in addition to their diagnosis of ASD (16.0% vs. 12.5%, *p*: 0.005). Similarly, those with comorbid GDD also had a statistically significant increased likelihood of medical comorbidity (34.9% vs. 18.2%, *p*: < 0.001).

Subjects were then divided based on sex and compared based on the presence/absence of a specific category of medical comorbidity. There was no sex difference between those with and without medical comorbidity (boys = 77.1 vs. 78.5, *p*: 0.52). However, when we broke down medical comorbidity into its categories, girls were more likely to have neurological comorbidities and/or genetic comorbidities (14.8% vs. 9.64%, *p*: 0.009 and 4.92% vs. 2.75%, *p*: 0.04). There was no statistically significant sex difference in the other categories.

## 4. Discussion

Our study found that medical comorbidities existed in a third of children diagnosed with ASD at the primary center for ASD in Manitoba during the time frame reviewed. This is consistent with other studies that have noted rates of medical comorbidities in children diagnosed with ASD that are higher than baseline rates [8,9,13,14,15]. Rates of comorbidities in other studies have varied based on the type of comorbidities included but range from about 10 to 77% [13,16].

The most observed category of comorbidity among our population, with an overall rate of 10.8%, was neurological comorbidities. Other studies have found similar rates of neurologic conditions in individuals with ASD, ranging from 1.1 to 14.2% [17].

There were children in our study who were noted to have hypotonia and/or early motor delays as a medical comorbidity. A link between hypotonia and ASD has been previously reported, and hypotonia has been proposed as a “red flag” symptom that should prompt closer monitoring and/or assessment for ASD [18,19]. There is also reasonable consensus that there is an association between early motor delays (in toddlerhood) and future diagnosis of ASD [20]. Interestingly, there were several individuals with plagiocephaly or brachycephaly in our study. Positional plagiocephaly does appear to be associated with hypotonia and/or delayed motor skills [21]. It stands to reason, therefore, that plagio-/brachycephaly can also be a “red flag” symptom in young infants. Further research in this area is warranted.

There were also children in our study who were noted to have cerebral palsy as a medical comorbidity. Children with cerebral palsy are known to be at a higher risk of ASD, with rates between 4.7 and 18.4% depending on the CP type [22]. Certainly, as others have highlighted, these rates support routine screening for symptoms of ASD in young children with cerebral palsy with a low threshold for full ASD assessment [22,23,24]. With ASD rates that are double or more than the general prevalence, it is plausible that there is an etiologic link between CP and ASD [23]. Brain injury location has been proposed as a possible factor in determining if an individual with CP will have comorbid ASD—with ASD being more associated with white matter injury [25]. Intriguingly, this may suggest that there can be a role for neuroimaging in ASD diagnosis of some specific patients in the future, especially with advances in neuroimaging technology.

A history of seizure/seizures, both febrile and afebrile, was a common medical comorbidity in our study. ASD prevalence in children with epilepsy has been shown to be elevated compared with baseline ASD prevalence, ranging from 4.7% in those with general epilepsy to 47.4% in those with Dravet syndrome [26]. Similarly, ASD is a risk factor for epilepsy, which has been reported in 8–30% of patients with ASD [27], with higher seizure rates existing in girls, those with comorbid intellectual developmental disability (IDD) and severe language dysfunction compared with those with higher-functioning autism [27]. These findings support a relationship between these two conditions, warranting close monitoring for ASD in children with epilepsy and vice versa.

There were several children with a history of stroke in our study. Children who suffer ischemic strokes have been shown to have a 2.6-fold increase in ASD (in childhood ischemic stroke without accompanying seizure disorder and/or cerebral palsy—independent risk factors for ASD themselves) [28,29]. Perinatal stroke has been associated with an 11.4% prevalence of ASD [30]. While stroke in pediatric patients is rare, similarly to patients with a history of cerebral palsy and seizure disorder, these findings support routine screening for ASD symptoms in any child with a history of stroke.

High rates of other comorbidities existed in our population, including common comorbidities in the categories of allergic, GI, and ENT conditions. These included allergies, asthma, and eczema, as well as conductive hearing loss, recurrent acute otitis media, insertion of tympanostomy tubes, difficulties in feeding, and iron deficiency. Other studies have shown that these common childhood diseases are common in children with ASD [14]. There are several factors that may cause children with ASD to be more challenging to diagnose with common allergic and/or GI conditions, including repetitive or atypical behaviors and limited language skills. Sensory or behavioral symptoms that are seen in many children with ASD may mask a child’s ability to communicate physical symptoms such as abdominal pain or pruritis; therefore, children with ASD may have common diseases that are more difficult to detect/diagnose and hence treat than children without ASD [14]. Our study would suggest that physicians who work in the areas of allergy, GI, and ENT should be aware of the diagnostic features of ASD, have a high index of suspicion for ASD, and should have a low threshold to assess for ASD or refer patients to be assessed for ASD.

In our study, medical comorbidities were more likely to exist in two specific groups: premature infants and those with global developmental delay.

Children who were prematurely born were more likely to have medical comorbidities in addition to their diagnosis of ASD. This result is not altogether surprising, knowing what we know about premature children and the higher rates of medical complexity that exists within this population at baseline [31]. In our modern world, children are becoming more medically complex as advancements in neonatology and pediatrics care allow for better outcomes for children prematurely born and/or with medical comorbidities. Rates of ASD are higher in premature infants, with reported prevalence rates of 7–13.2% in premature infants born <37 weeks gestational age [32,33] and 15.7% in those born <29 weeks gestational age [34]. Some studies have suggested that ASD features in children born extremely prematurely present differently from their term-born peers with ASD, especially in those born extremely prematurely [35]. Features such as impaired social communication, sensory sensitivities, poor motor skills, and overall severity of ASD have been demonstrated to be more significant in premature children with ASD [36,37]. Our study would suggest that this group is also different from term-born children with ASD due to increased medical comorbidities. Interestingly, increased neuroinflammation in the premature infant has been identified as a possible factor for increased rates of ASD in this group [36]. Inflammation plays a role in many prematurity-related comorbidities such as bronchopulmonary dysplasia [38], retinopathy of prematurity [39], and intraventricular hemorrhage [40]. This intriguing idea is important as it can mean that with further research, the presence of (certain) medical comorbidities may be able to act as a clue in the identification of children in this group who have increased ASD risk, allowing providers to outline child-specific developmental information for the parents of children who are born preterm.

Our study also found that children with comorbid GDD were also more likely to have medical comorbidities in addition to their diagnosis of ASD. This may, in part, reflect comorbidities that themselves have increased prevalence of GDD or IDD, e.g., cerebral palsy, trisomy 21, and epilepsy. GDD is a common comorbidity among children with ASD [41]; however, rates of IDD are variable [12]. Differentiating between GDD that related to a medical comorbidity, for example, a genetic syndrome, may allow us to better understand the expected pattern of development.

In our study, girls were found to be more likely to have a neurological comorbidity in addition to their diagnosis of ASD. Girls were also found to be more likely to have a genetic comorbidity in addition to their diagnosis of ASD. As girls with ASD tend to present with more subtle symptoms [1], this finding is important, as the existence of medical comorbidities in girls should lead to a high index of suspicion for ASD.

Importantly, the results of our study demonstrated that the presence or absence of medical comorbidities did not affect the use of the ADOS-2 diagnostic tool.

Similarly, the presence or absence of medical comorbidities did not affect the age of referral or the age of diagnosis. This would indicate that children are receiving equal access to diagnosis regardless of the presence or absence of medical comorbidities.

In our study, rates of comorbid ADHD or suspected ADHD did not differ among those with ASD with and without medical comorbidities, suggesting no correlation between having ADHD and other medical comorbidities. ADHD has been shown to be one of the most common comorbidities in individuals diagnosed with ASD. In our study, ADHD/suspected ADHD was only reported in 107 (5.76%), which is much lower than rates of 20–30% that have been shown in other studies [41,42]. One explanation of this can be that our study looked at ASD in preschool children, many of whom may go on to have a diagnosis of ADHD in the future, but who would not yet meet criteria at the time of their ASD diagnosis.

The heterogeneity of ASD has been a point of interesting debate. A significant consideration in heterogeneity is appropriate prognosis and support planning. There are important differences between what will be helpful and/or required to support an individual with ASD who has typical cognitive and language skills and an individual who is non-verbal and has intellectual developmental disorder in addition to their diagnosis of ASD [5].

The needs of children with neurological comorbidities, genetic syndromes, a history of prematurity and prematurity-related medical comorbidities, and global developmental delay may be different than those of healthy children. Children with these types of comorbidities with ASD and children with ASD who are otherwise healthy also present differently. Perhaps the categorization of ASD as primary vs. secondary is one way of highlighting the additional complexities of medically complicated children with ASD. Specifically, we suggest that primary ASD would be the diagnosis made in an otherwise healthy child, and secondary ASD would be made in a child who meets the criteria for a diagnosis of ASD but who also presents with multiple medical comorbidities such as extreme prematurity, stroke and/or cerebral palsy, and genetic or syndromic conditions with accompanying global developmental delay.

Many medical diagnoses are categorized into primary and secondary based on the underlying etiology of the disease. Neurodevelopmental disorders are not typically delineated in this fashion; however, our study highlights that there is a subset of individuals diagnosed with ASD who are complex, and perhaps separating these patients from those with “simple ASD” may be helpful for prognostication, allocation of resources, and intervention/therapy planning.

Our study was limited in its ability to assess the socioeconomic status. We used parental education level as a substitute measure. Our study found that children whose parents had less than a high school education were more likely to have medical comorbidities in addition to their diagnosis of ASD. Lower SES has been shown in some studies to be associated with higher ASD prevalence, as well as higher rates of medical comorbidities [33].

This was a retrospective study that led to several key limitations. When examined in single-predictor analyses, we found that higher rates of medical comorbidities were significantly associated with prematurity, global developmental delay, and less than high school education. In a multiple-predictor logistic regression analysis, which included previously significant variables, only global developmental delay was found to be a significant predictor of medical comorbidities (see Table 1). This tells us that our variables are interrelated. Unfortunately, our study was not powered or set up to study these variables as modifiers to each other, something that we hope to explore in future studies. Another important limitation of our study was that the severity of ASD (Level 1, 2, or 3) was not consistently noted in the charts reviewed, largely as the children studied are preschool. This limits our ability to interpret how our results are affected by differences in ASD severity. It is also impossible to know in a retrospective chart review if the diagnosis of ASD led to the diagnosis of medical comorbidities or vice versa. As such, we are unable to label any of our findings as “risk factors” for ASD and can only speak to associations between characteristics and increased ASD rates. Missing data is a huge limitation of our study. We did not consistently have all possible diagnostic testing that was performed on the children in our study (including those carried out pre- and post-natally). Similarly, constipation and picky/restricted eating have both been noted as an important common GI condition in patients with ASD [9,13,14,15]. Unfortunately, our study did not consistently identify these important comorbidities. One explanation for this is that reporting of medical comorbidities such as constipation, picky eating, and other comorbidities would depend on if a clinician felt that the severity of the condition met the threshold for a medical comorbidity rather than a behavioral challenge. In our study, parental education was used as a substitute for socioeconomic status as household income was not collected; however, parental education was missing in 803 charts (43.2%). Missing data equally existed between individuals with and without medical comorbidities (411 missing had no medical comorbidities, and 395 had medical comorbidities); therefore, we felt that it was reasonable to analyze this variable while recognizing that missing data may have affected the results.

## 5. Conclusions

Medical comorbidities exist in children with ASD at high rates. These rates were particularly high in children prematurely born and those with comorbid GDD.

ASD is a heterogeneous neurodevelopmental disorder. Some of this heterogeneity appears to exist due to differences between individuals with and without medical comorbidities, especially comorbidities that affect a child’s overall function and presentation, such as prematurity, epilepsy, stroke, cerebral palsy, and genetic or syndromic conditions with or without accompanying global developmental delay.

We suggest that categorizing ASD into primary and secondary can help with prognostication, service allocation decisions, and the ability to provide appropriate resources to all patients with ASD.

## Figures and Tables

**Table 1:** Differences in demographics and clinical presentation between children with medical comorbidity and those without medical comorbidity.

	All (N = 1858)	Comorbidity Yes (N = 539)	Comorbidity No (N = 1319)	*p* Value	*p* Value (LR ^1^)
Mean maternal age at birth of child	30.1 (SD = 6.04)	30.3 (SD = 6.27)	30 (SD = 5.9)	0.32	-
<High school education	136	51 (18.0%)	85 (11.1%)	0.003	0.850
Male sex	1452 (78.1%)	416 (77.1%)	1036 (78.5%)	0.52	-
Premature	251 (13.5%)	86 (16.0%)	165 (12.5%)	0.005	0.125
Mean age of child at referral	2.77 (SD = 1.03)	2.75 (SD = 1.17)	2.8 (SD = 0.99)	0.33	-
Mean age of child at diagnosis	3.81 (SD = 1.12)	3.85 (SD = 1.23)	3.81 (SD = 1.11)	0.44	-
ADOS-2 tool used in diagnosis	603 (32.5%)	187 (34.7%)	416 (31.5%)	0.19	-
GDD diagnosis	428 (23.0%)	188 (34.9%)	240 (18.2%)	<0.001	<0.001
ADHD diagnosis or suspected	107 (5.76%)	34 (6.30%)	73 (5.53%)	0.65	-

^1^: LR—logistic regression model.

**Table 2:** Comorbidities.

Comorbidity	N (%)
**All Comorbidities**	**539 (100%)**
**Neurological Conditions**	**200 (37.0%)**
Cerebral palsy	9 (1.67%)
Delayed motor milestones	3 (0.56%)
Febrile seizures	26 (4.82%)
Head injury	6 (1.11%)
Hypertonia	2 (0.37%)
Hypotonia	31 (5.75%)
Hypoxic ischemic encephalopathy	3 (0.56%)
Infantile spasms	6 (1.11%)
Intraventricular hemorrhage	3 (0.56%)
In utero exposure to substance(s)	8 (1.48%)
Macrocephaly	13 (2.41)
Meningitis	4 (0.74%)
Microcephaly	6 (1.11%)
Seizures	42 (7.79%)
Sensorineural hearing loss	5 (0.93%)
Septo-optic dysplasia	5 (0.93%)
Staring spells NYD ^1^	2 (0.37%)
Stroke—perinatal or childhood	5 (0.93%)
**Genetic Conditions**	**60 (11.2%)**
Abnormal genetic results/variants NOS ^2^	6 (1.11%)
Chromosomal abnormalities unspecified	12 (2.23%)
Klinefelter syndrome	4 (0.74%)
Duane syndrome	2 (0.37%)
Trisomy 21	4 (0.74%)
Tuberous sclerosis (diagnosed/suspected)	2 (0.37%)
**Congenital Non-Syndromic Conditions**	**18 (3.35%)**
Craniosynostosis	5 (0.93%)
Cleft lip and/or palate	5 (0.92%)
Congenital cytomegalovirus (CMV)	2 (0.37%)
**Allergic Conditions**	**151 (28.0%)**
Allergy/allergies	65 (12.1%)
Asthma	54 (10.0%)
Eczema	68 (12.6%)
**Gastrointestinal Conditions**	**29 (5.39%)**
Bowel obstruction/perforation	2 (0.37%)
Feeding difficulties	4 (0.74%)
Food protein-induced enterocolitis syndrome	3 (0.56%)
Gastroesophageal reflux	3 (0.56%)
Iron deficiency	2 (0.37%)
Overweight/obese	3 (0.56%)
Pica	3 (0.56%)
Pyloric stenosis	2 (0.37%)
**ENT Conditions**	**40 (7.43%)**
Conductive hearing loss	10 (1.86%)
Recurrent/frequent acute otitis media	5 (0.93%)
Tonsillectomy and/or adenoidectomy	4 (0.74%)
Tympanostomy tubes inserted	18 (3.34%)
**Other**	**170 (31.6%)**
Ambiguous genitalia	2 (0.37%)
Astigmatism	9 (1.67%)
Brachycephaly	7 (1.30%)
Bronchopulmonary dysplasia	4 (0.74%)
Dental caries/required dental surgery	3 (0.56%)
Hemangioma	2 (0.37%)
Heart murmur NOS	3 (0.56%)
Nystagmus	4 (0.74%)
Plagiocephaly and/or torticollis	32 (5.94%)
Recurrent/severe pneumonia	11 (2.04%)
Retinopathy of prematurity	4 (0.74%)
Sickle cell disease	3 (0.56%)
Strabismus/esotropia	32 (5.94%)
Structural heart defects	5 (0.93%)
Toe walking NOS	2 (0.37%)
Traumatic injury (burns or fractures)	8 (1.48%)
Wears glasses	38 (7.05%)

^1^: NYD—not yet determined; ^2^: NOS—not otherwise specified.
